# The Magnetic Search Coil (MSC) on the TRACERS Mission

**DOI:** 10.1007/s11214-025-01200-7

**Published:** 2025-08-05

**Authors:** G. B. Hospodarsky, A. J. Carton, R. T. Dvorsky, D. L. Kirchner, D. M. Miles, S. R. Bounds, I. W. Christopher, D. Crawford, K. Deasy, J. S. Dolan, J. B. Faden, G. W. Fessenden, C. Hansen, R. L. Helland, S. D. Klinkhammer, M. C. Miller, K. J. Morris, C. W. Piker, O. Santolik, K. Steele, T. A. Tompkins, M. D. Webb, D. Wilkinson

**Affiliations:** 1 Department of Physics and Astronomy, University of Iowa, 203 Van Allen Hall, Iowa City, 52242 IA USA; 2 Department of Space Physics, Institute of Atmospheric Physics of the Czech Academy of Sciences, Prague, Czechia; 3 Faculty of Mathematics and Physics, Charles University, Prague, Czechia

**Keywords:** Magnetosphere, Magnetic reconnection, Waves, TRACERS mission, Search coil

## Abstract

The Magnetic Search Coil (MSC) instruments on the TRACERS mission provide the three magnetic components of the waves from ∼1 Hz to 1 kHz from two closely spaced spacecraft in low Earth orbit that pass through the magnetospheric cusp. These measurements of Alfvén and other waves help meet the TRACERS Science Objective 3: “Determine to what extent dynamic structures in the cusp are associated with temporal versus spatial reconnection”. The TRACERS MSC uses a three axis, dual sensor coil system and amplifiers with current feedback to provide continuous analog outputs to the Electric Field Instrument (EFI) Electric Signal Processing (ESP) Board. The ESP digitally samples each MSC analog output channel with 16-bit resolution at 2048 samples/second and sends the digitally sampled data to the Central Data Processing Unit (CDPU). The TRACERS MSC design, calibration, and performance is described.

## Introduction

The Tandem Reconnection and Cusp Electrodynamics Reconnaissance Satellites (TRACERS) is a Small Explorer-class NASA mission with the main science goal: Connecting the cusp to the magnetosphere by discovering how spatial or temporal variations in magnetic reconnection drive cusp dynamics (Miles et al. 2025a). Reconnection at Earth’s magnetopause is believed to be driven by the solar wind dynamic pressure on the dayside and is believed to be continuously occurring somewhere on the magnetopause (Cassak and Fuselier 2016). However, the observation of ion steps in the cusp indicates reconnection variability, either temporally sporadic from a fixed spatial location (temporal reconnection), or temporally steady from a variety of spatial locations (spatial reconnection), or some combination of the two. To address this science goal, the TRACERS mission consists of two closely spaced, identical spacecraft, in a low altitude circular Sun-synchronous Earth orbit that passes through the northern magnetospheric cusp (Petrinec et al. 2025). The TRACERS Instrument Suite (TIS) consists of: the Analyzer for Cusp Electrons (ACE) (Halekas et al. 2025), the Analyzer for Cusp Ions (ACI) (Fuselier et al. 2025), the Electric Field Instrument (EFI) (Bonnell et al. 2025), the Fluxgate Magnetometer (MAG) Instrument (Strangeway et al. 2025), the Magnetic Search Coil (MSC), the MAGnetometers for Innovation and Capability (MAGIC) Fluxgate Magnetometer Technology Demonstration (Miles et al. 2025b), and a common Main Electronics Box (MEB) that contains the Low Voltage Power Supply (LVPS) and the Common Data Processing Unit (CDPU). This paper describes the two MSC instruments, including their scientific objectives, their design and fabrication, and their testing and calibrations.

### Science Objectives

Miles et al. (2025a) discusses in detail the overall Science Objectives of the TRACERS mission. The MSC instruments are included on the science payload to specifically help address Science Objective #3: “Determine to what extent dynamic structures in the cusp are associated with temporal versus spatial reconnection”. Ion outflows, electron precipitation, Alfvén and other waves, are several of the dynamic structures or features observed in the cusp associated with temporal versus spatial reconnection. Based on previous observations and theory (Chaston et al. 2000, 2007, 1999, 2006; Chen et al. 2005; Kletzing 1994; Kletzing and Hu 2001; Miyake et al. 2003; Tanaka et al. 2005), spatial reconnection events should have a distinct set of dynamic features associated with them while temporal events should have a different set and sequence of features. Primarily, where these waves occur relative to cusp ion steps determines if they are associated with temporal or spatial reconnection. For temporal reconnection, it is expected that time dispersed electrons, followed by Alfvén waves, followed by cusp ions will be detected. This sequence should occur independent of IMF orientation and dynamic pressure since the temporal opening of field lines generates the waves. For spatial reconnection, no Alfvén waves and time-dispersed electrons are expected. However, current structures at cusp ion step edges, and unstable wave modes associated with particle and energy release from the reconnection are possible. By separating the spatial and temporal cusp ion signatures, TRACERS will confirm that spatial and temporal events have different wave and particle signatures. The MSC will measure the wave magnetic fields to identify these waves.

### Requirements

The measurement requirements of the MSC were determined by the need to measure the waves that are likely to be detected in the cusp that are related to spatial and temporal reconnection as discussed above. For example, the MSC magnetic field measurements must determine wave propagation directions up to several hundred Hz to distinguish Alfvén waves from other wave modes. See Trattner et al. (2025) for a discussion on some of the wave modes expected to be encountered by TRACERS. To accomplish this, MSC measures the three components of the fluctuating magnetic field with sufficient sensitivity, dynamic range, and time resolution to capture the different types of waves (see Table 1 for a summary of the MSC performance parameters).

**Table 1:** MSC Parameters and Performance

Parameter	Performance
Field of View	3 axis measurements
Frequency Range	∼1 Hz to 1000 Hz
Sampling Rate	2048 16-bit samples/sec
Sensitivity	∼10^−6^ nT^2^/Hz @10 Hz
	∼10^−8^ nT^2^/Hz @100 Hz
Sensor/core length	15 cm
Sensor Alignment	<1^o^
Mass	0.7 kg
Power	∼0.8 W (nominal)
Max Spacecraft Spin Rate (signal from Earth’s B)	<30 rpm

### Heritage

The TRACERS MSC has direct heritage to the University of Iowa search coils provided for the ISEE (Gurnett et al. 1978; Scarf et al. 1978), Wind (Bougeret et al. 1995), Juno (Kurth et al. 2017), Van Allen Probes (Kletzing et al. 2013) and VIPER (sounding rocket) missions. The design and construction of the search coil for the VIPER sounding rocket which occurred early in the TRACERS project was especially valuable, in that it served as a precursor for the TRACERS MSC design and assembly.

## MSC Instrument Description

### Overview

**Fig. 1:**
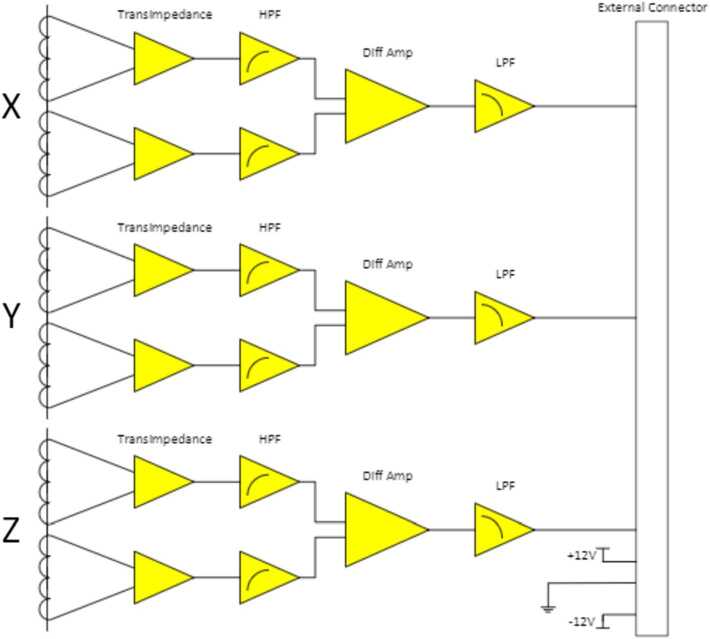
TRACERS MSC Block Diagram

**Fig. 2:**
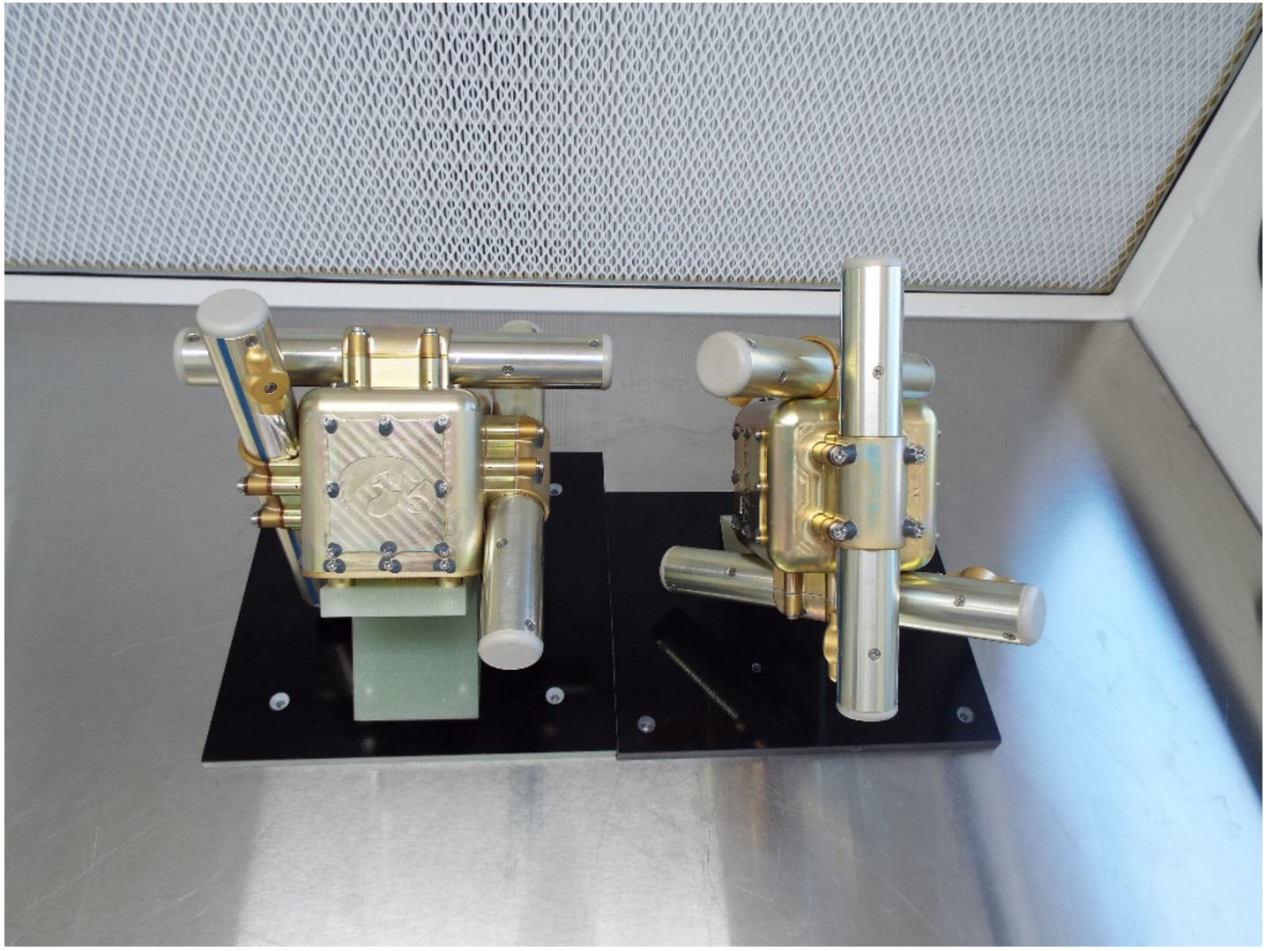
Picture of the two MSC units

The TRACERS MSC is based on the standard search coil design of thousands to tens of thousands of turns of wire on a high permeability core attached to an electric amplifier (Hospodarsky 2016). Figure 1 shows a block diagram of the TRACERS MSC and Fig. 2 shows a picture of the two completed MSC units mounted on test fixtures taken before delivery to the spacecraft. The two MSC units are nearly identical, each roughly the size of a 15.5 × 15.5 × 15.5 cm cube, and each weighing just under 700 grams. The MSC electronics are located in the center housing with the three sensor tubes mounted perpendicular to each other in a triaxial configuration to provide full 3D measurements of the wave magnetic fields. The <1 degree axis alignment was determined by analysis of the worst-case alignment tolerances of the mechanical parts making up the sensor axis. This analysis showed that the worst-case alignment error was 0.738 degrees. Power (+/−12 Volts) is provided from the LVPS (via EFI). The MSC continuously provides three analog signal outputs to the EFI Signal Processing Board (ESP) which are converted to digitized waveforms of 16-bit samples (+/−12 V dynamic range, 0.023 mV/ADC) at 2048 samples/second for each axis (see Bonnell et al. 2025, for a detailed discussion of this process). These digitized waveforms are sent to the CDPU which controls the amount of data saved and sent to the ground. The MSC is mounted on rigid bracket ∼0.65 meters from the spacecraft surface to help mitigate any spacecraft noise sources, opposite a similar bracket with the MAG and MAGIC sensors. Two of the MSC sensor coils are oriented parallel to the two EFI electric dipole antennas and the third parallel to the spacecraft spin axis. Each individual components of the MSC will be discussed in more detail below.

### MSC Sensor Design

**Fig. 3:**
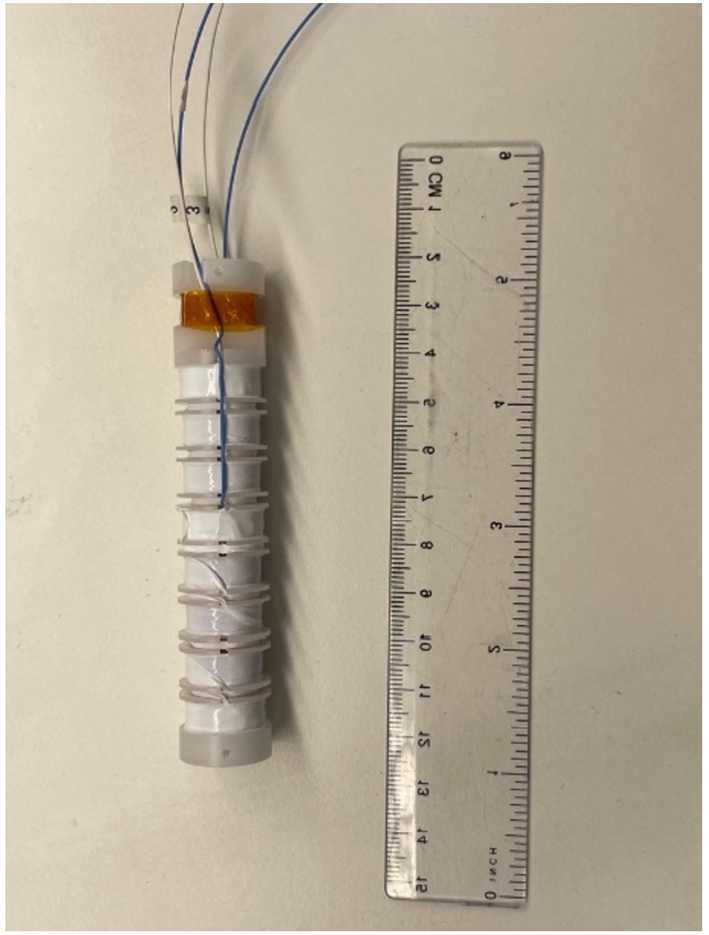
TRACERS sensor coil after winding

**Fig. 4:**
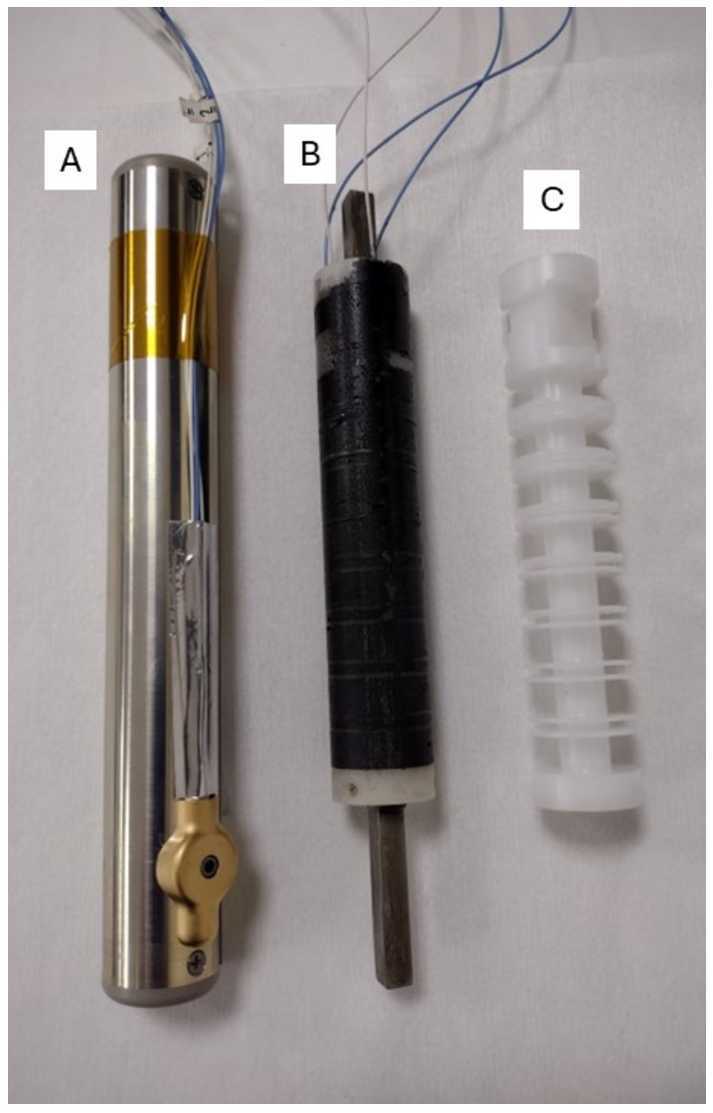
Picture of the TRACERS MSC Sensors in various steps of their assembly. An unwound sensor bobbin is shown in Fig. 4C. A wound and potted sensor coil with core is shown in Fig. 4B. Figure 4A shows a TRACERS sensor core in its electrostatic shield tube ready to be attached to the main MSC housing

The TRACERS MSC employs a dual sensor coil design with each axis sensor containing two coils of 26,000 turns of #44 AWG magnet wire wound on a Delrin bobbin (see Fig. 3 and 4C). The sensor coils were manufactured and wound at the University of Iowa. After winding, the bobbins were potted with an epoxy and the cores were installed (see Fig. 4B). Each sensor coil utilizes a mu-metal core approximately 15 cm in length (same length and material as used in the Juno search coil). The core length of 15 cm was chosen early in the TRACERS project to allow the MSC to fit in the possible launch vehicle faring envelopes being considered and still meet the sensitivity requirements to achieve the science goals. A thermal cycle test was performed on the assembled coils and cores to verify functionality and stability (coil resistance and inductance) over the temperature range the MSC will experience in flight. After the thermal cycle test, the coils were placed in their aluminum tubes (Fig. 4A), attached to the MSC main housing, and the coils soldered to the electronic board. The sensor tubes provide both a mechanical and an electrostatic shield for the sensor coils. To prevent any conductive path (e.g. a shorting turn) around the sensor axis that could reduce the sensitivity of the unit, each tube contains a non-conductive slot along the length of the tube (the blue stripe in Fig. 2), the tube mounting assemblies include nonconductive spacers, and the end caps are constructed with nonconductive materials.

### MSC Electronic Design

**Fig. 5:**
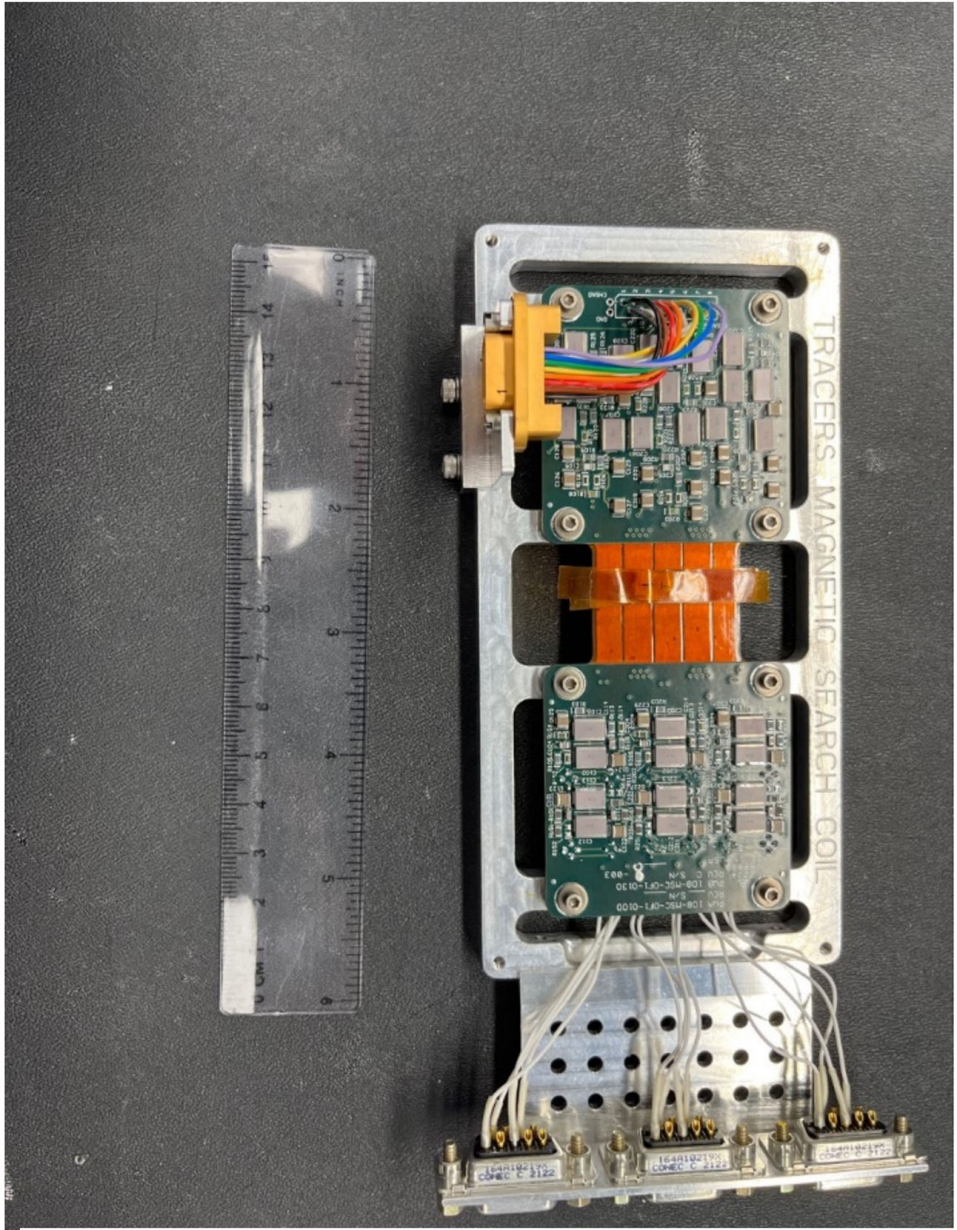
The MSC Electronics

**Fig. 6:**
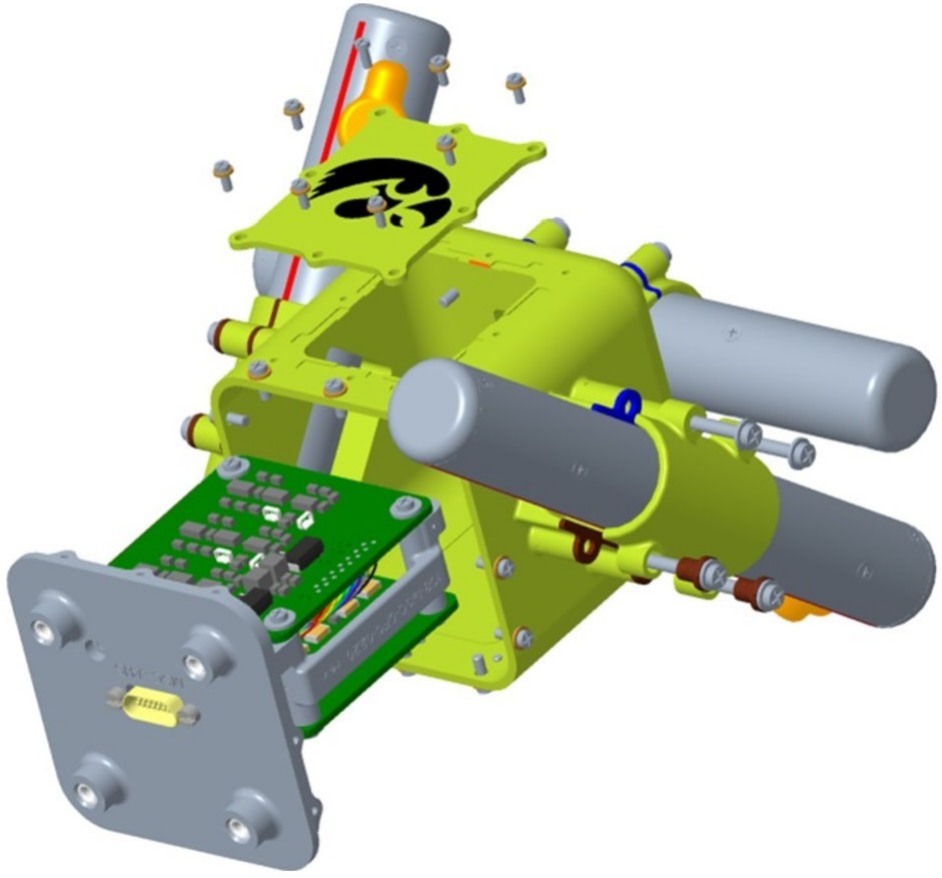
Exploded view of the MSC assembly

The MSC instrument electronics are constructed on a rigid-flex electronic assembly shown in its flat configuration in Fig. 5. The rigid-flex interconnect design was chosen to save room on the rigid boards, and to reduce the complexity and work of connecting the two boards. The two rigid boards are approximately 5 × 5 cm in size. When installed into the MSC housing, the assembly is folded with the two rigid boards mounted parallel to each other as shown in Fig. 6. The sensor coil outputs (2 per sensor axis) operate in current mode, so each go to a transimpedance amplifier with current feedback to flatten the voltage response over frequency. The voltage output of this amplifier then goes through a high pass filter to reduce the signal induced by the spacecraft spinning in the Earth’s DC magnetic field. The signals are then combined in a differential amplifier to reduce thermal noise and increase bandwidth and then passed through a low pass antialiasing filter before being digitized by the EFI ESP board. The voltage response is flattened over frequency to provide a more easily calibrated waveform output (see Sect. 3). The MSC electronics are powered by +/−12 Volts supplied from the LVPS and includes input power filtering. The nominal power draw is ∼0.8 Watt.

### Mechanical Design and Assembly

The TRACERS MSC mechanical design was loosely based on the Van Allen Probes EMFISIS search coil (Kletzing et al. 2013) with the electronics located in a central housing and the sensor coils mounted to this housing. The overall size of the TRACERS MSC units was driven by the need to fit in the smallest possible launch vehicle faring envelopes being considered early in the TRACERS project. The University of Iowa provided a search coil for the VIPER sounding rocket (launched May 2021) very early in the TRACERS project and it was decided to use very similar mechanical designs for the two mission to allow the VIPER search coil to be a pathfinder for the TRACERS design and assembly. This early VIPER search coil design and build greatly increased the efficiency of the TRACERS MSC assembly. Figure 6 shows an exploded view of the MSC components and Fig. 2 shows the actual assembled MSC units.

#### Thermal Design

MSC is thermally isolated from the spacecraft and bracket, relying solely on passive thermal control via its dedicated MLI thermal blanket. Minor conductive coupling through the harness is negligible due to the MSC’s low power dissipation and benign thermal environment. The MSC is designed to operate over a large temperature range, and has been calibrated between −30 and +40 °C, with survival temperature tested to +/−50 °C. The predicted operational temperature range of the MSC using the most recent thermal modeling based on the MSC distance from the spacecraft body, the power dissipation, and TRACERS planned orbit is −6 to +17 °C.

## MSC Testing and Calibration

Both MSC instruments went through a program of performance, calibration and qualifications tests, first at the component and instrument level, and then at the integrated suite level, primarily employing a number of calibration coils and magnetic shields available at the University of Iowa. A summary of these tests is discussed below.

### Component and Instrument Testing and Calibration

**Fig. 7:**
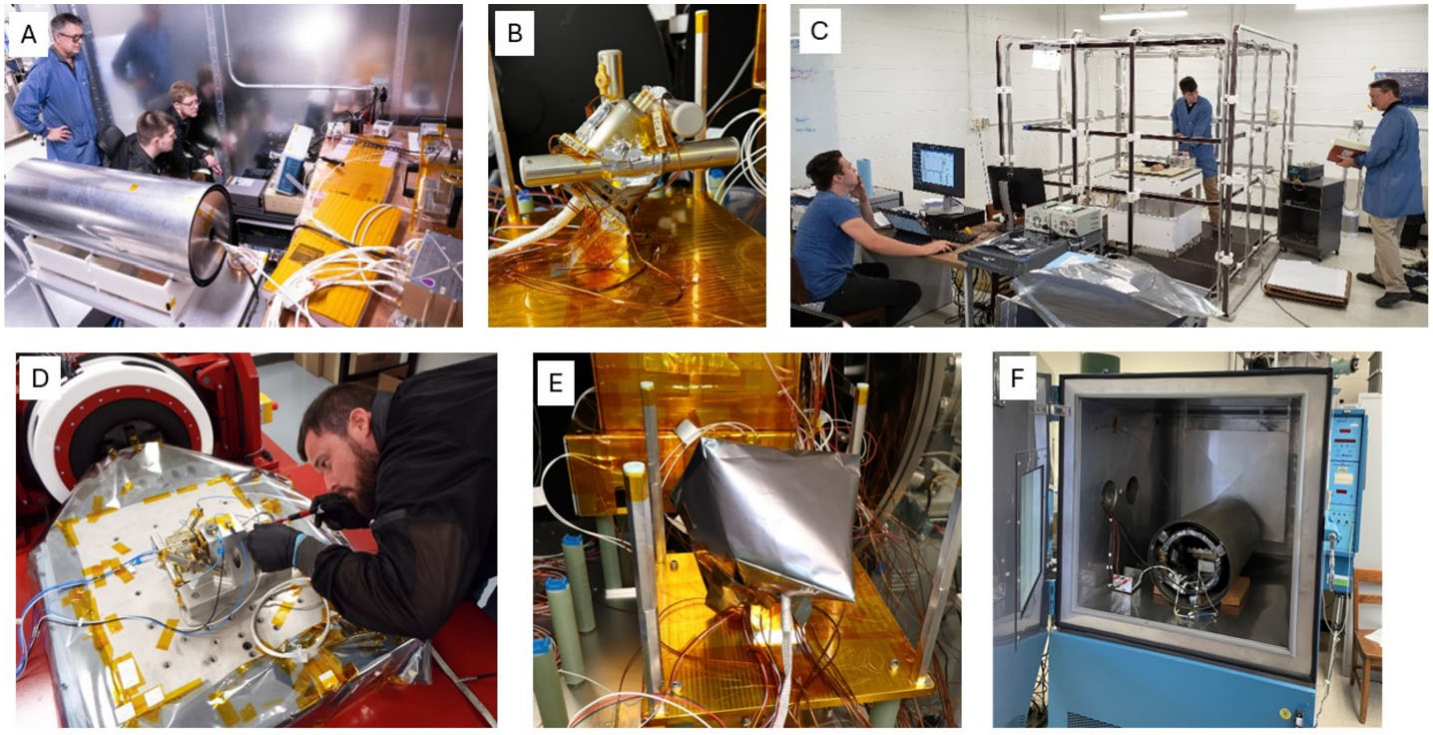
Pictures of MSC EMI/EMC (A), Thermal Vacuum (B and E), Merritt Coil (C), Vibration (D), and Thermal (F) Testing

Both the sensor coils and the electronics were tested individually before being assembled into the complete MSC unit to verify functionality and performance. After assembly of the sensors and electronics into the tri-axial configuration (Fig. 2 and 6), the units were retested, and instrument level calibrations were performed. These tests and calibrations primarily involved using solenoid calibration coils inside a cylindrical mu-metal shield (reduces 60 Hz and other lab interference) to produce a known magnetic field over the frequency range of the MSC (∼1 Hz to 1 kHz). Figure 7A and 7F shows some of the mu-metal shield cans used in the MSC testing. Additional tests were performed using a Merritt Coil System at the University of Iowa to simulate the MSC performance on a spinning TRACERS spacecraft in Earth’s magnetic field (Fig. 7C).

**Fig. 8:**
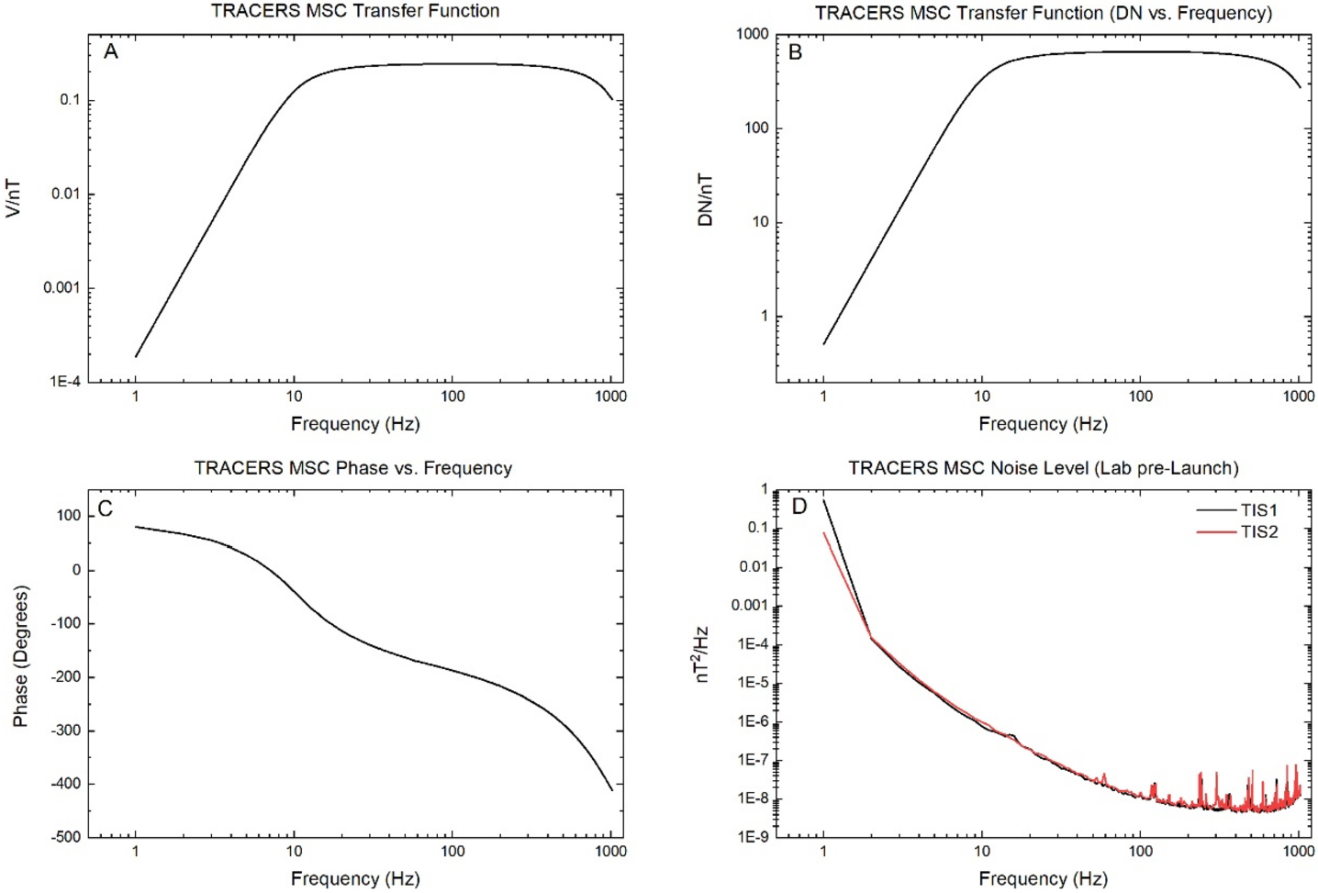
The TRACERS MSC Transfer Functions (8A and 8B), Phase shift compared to signal to calibration coil over frequency (8C), and the Noise Level measured in the lab with the fully integrated Instrument Suite before launch. (8D)

The calibrations of the MSC were determined in a two-step process. Initially, a detailed amplitude and phase calibration of each axis of the MSC units was performed using different solenoid calibration coils inside cylindrical mu-metal shields to produce a magnetic field with a known magnitude. The resulting data relate input field strength to the voltage at the output of the MSC over the frequency range of the sensor (∼1 Hz to 1 kHz). These calibrations were performed at room temperature (∼+22 °C), and at the possible maximum temperature extremes of the sensors (−30 and +40 °C). The temperature calibrations were performed in large thermal chambers at the University of Iowa that can contain the calibration coils and the mu-metal shield cans (see Fig. 7F). The second calibration step relates the voltage input to the EFI ESP and the resulting output telemetry value (data number (dN)) to the CDPU. After integration of the TRACERS Instrument Suite (TIS) for each spacecraft, end-to-end calibrations of MSC and the MSC EFI ESP telemetry output were performed by driving the calibration coil to produce a known magnetic field strength and verifying the telemetry values out of CDPU. Figure 8A and 8B show the resulting transfer functions of the Y channel of MSC FM1, both in voltage out of the MSC unit (Fig. 8A) and the telemetry value out of the EFI ESP (Fig. 8B) for a 1 nT input magnetic field over the frequency range of the MSC. As can be seen, the frequency response is nearly flat over the range of about 10 Hz to 600 Hz, which provides a more easily calibrated waveform output in the prime measurement region. The transfer function in this frequency range is about 0.24 V/nT, or about 652.5 dN/nT. Figure 8C shows the phase shift over frequency of the MSC output voltage compared to the input voltage to the calibration coil.

All three axis of both MSC units are well matched and have nearly identical transfer functions and phase response (within measurement error). Furthermore, the calibrations were found to be consistent over the temperature range the MSC units are expected to encounter in orbit, allowing a single calibration table to be used for both units as was done for the Van Allen Probes EMFISIS search coils. It is difficult to determine the absolute noise level of a search coil sensor until after launch. In the lab, various interference sources, primarily 60 Hz and harmonics due to power lines, produce wave magnetic fields much larger than typically observed on orbit. Shield cans can mitigate this interference but cannot totally remove it. In flight, low level interference from the spacecraft or another instrument may be detected that was not seen on the ground. To mitigate the possibility of the MSC detecting spacecraft or other instrument interference signals, TRACERS employed a Magnetic Control Plan that emphasized good design principles and screening of subsystems and components (Miles et al. 2025c). Figure 8C shows noise levels measured by both MSC units while in shield cans during a Comprehensive Performance Test (CPT) performed before the TIS was installed on the spacecraft. Both units have very similar sensitivity and noise levels with the small differences believed to be due to different lab environmental noise sources present on the day of the tests.

### Qualification Testing

The two MSC instruments went through a standard program of electromagnetic interference and compatibility (EMI/EMC) (Fig. 7A), vibration (Fig. 7D), and thermal vacuum testing (Fig. 7B and 7E). For instrument level EMI/EMC and thermal vacuum, the MSC was tested together with the entire TRACERS Instrument Suite except for ACI, allowing a very flight-like test of the instruments and flight harnesses, and the opportunity to investigate any noise sources from the other instruments. During thermal vacuum testing (Fig. 7B and 7E), no stimulus was provided for the MSC, so the ambient 60 Hz signals present in the lab/chamber was used to verify functionality of the MSC. For vibration testing, the MSC units were vibrated individually, and an aliveness test was performed between each axis vibration to verify continued functionality, with a full functional test performed after completion of all three axis vibrations to verify no change in performance.

## MSC Operation

The MSC instruments have a very simple operations plan and produces the same telemetry stream consisting of continuous, 3 axis waveforms at 2048 samples per second whenever it is on (same sampling rate as the EFI EAC channels (Bonnell et al. 2025). MSC does not have the capability to produce onboard data products, such as spectral matrix or spectra, so these products will be produced on the ground. The MSC requires no commands from the CDPU beyond powering the MSC electronics on or off. The nominal operation plan is for MSC to be on and collecting data during the entire orbit, with the CDPU controlling the amount of data saved and sent to the ground, avoiding power cycling and operational complexity. Housekeeping data for the MSC (secondary voltage and current) is sampled at the LVPS and monitored at the CDPU. During the Region of Interest (ROI) (see Miles et al. 2025a and Petrinec et al. 2025 for a discussion of the ROI region), full 3 axis MSC continuous waveforms will be sent to the ground for further processing. During the rest of the orbit, called the “back orbit”, to reduce data volume, a subset of the continuous collected MSC data will be decimated by the CDPU and sent to the ground (see Dorelli et al. 2025 for a discussion of the back orbit).

## Conclusions

The MSC instruments are part of the highly capable TRACERS Instrument Suites. providing the three magnetic components of the waves from ∼1 Hz to 1 kHz collected by two closely spaced spacecraft in low Earth orbit. The measurement of Alfvén and other waves in the cusp will help meet the TRACERS Science Objective 3: “Determine to what extent dynamic structures in the cusp are associated with temporal versus spatial reconnection”. In addition, MSC will make additional magnetic wave observations throughout the TRACERS orbit, potentially providing information about the auroral and polar cap regions, plus other areas of interest (Dorelli et al. 2025), and provide an opportunity to collaborate with other missions (Trattner et al. 2025).
